# Supplementary material for: Gene-environment interaction analysis incorporating sex, cardiometabolic diseases, and multiple deprivation index reveals novel genetic associations with COVID-19 severity
Source: medRxiv. 2021 Aug 23:2021.08.13.21261910. Preprint. [Version 1] doi: 10.1101/2021.08.13.21261910 (PMC8404897; doi:10.1101/2021.08.13.21261910)
Supplement: Supplement 1 [file media-1.docx]

**Gene-environment interaction analysis incorporating sex, cardiometabolic diseases, and multiple deprivation index reveals novel genetic associations with COVID-19 severity**

Kenneth E. Westerman*^1,2,3^, Joanna Lin*^1^, Magdalena Sevilla-Gonzalez^1,2,3^, Beza Tadess^1,2^, Casey Marchek^1,2^, Alisa K. Manning^1,2,3#^

*co-first authors

#corresponding author:

Alisa K. Manning

Clinical and Translational Epidemiology Unit

Massachusetts General Hospital

Boston, MA, 02114, USA

[amanning@broadinstitute.org](mailto:amanning@broadinstitute.org)

phone: 617-714-7662

fax: 617-800-1762

**Supplemental Figures**

**Supp. Fig. S1**: Severe COVID-19 phenotype definition flow chart. This definition is based on the “B2” phenotype used by the COVID-19 HGI group.

**Supp. Fig. S2**: Sex analysis joint and interaction analysis results. The Manhattan plot displays association test strengths for the joint (top panel) and interaction (bottom panel) tests as a function of genomic position (*x*-axis).

**
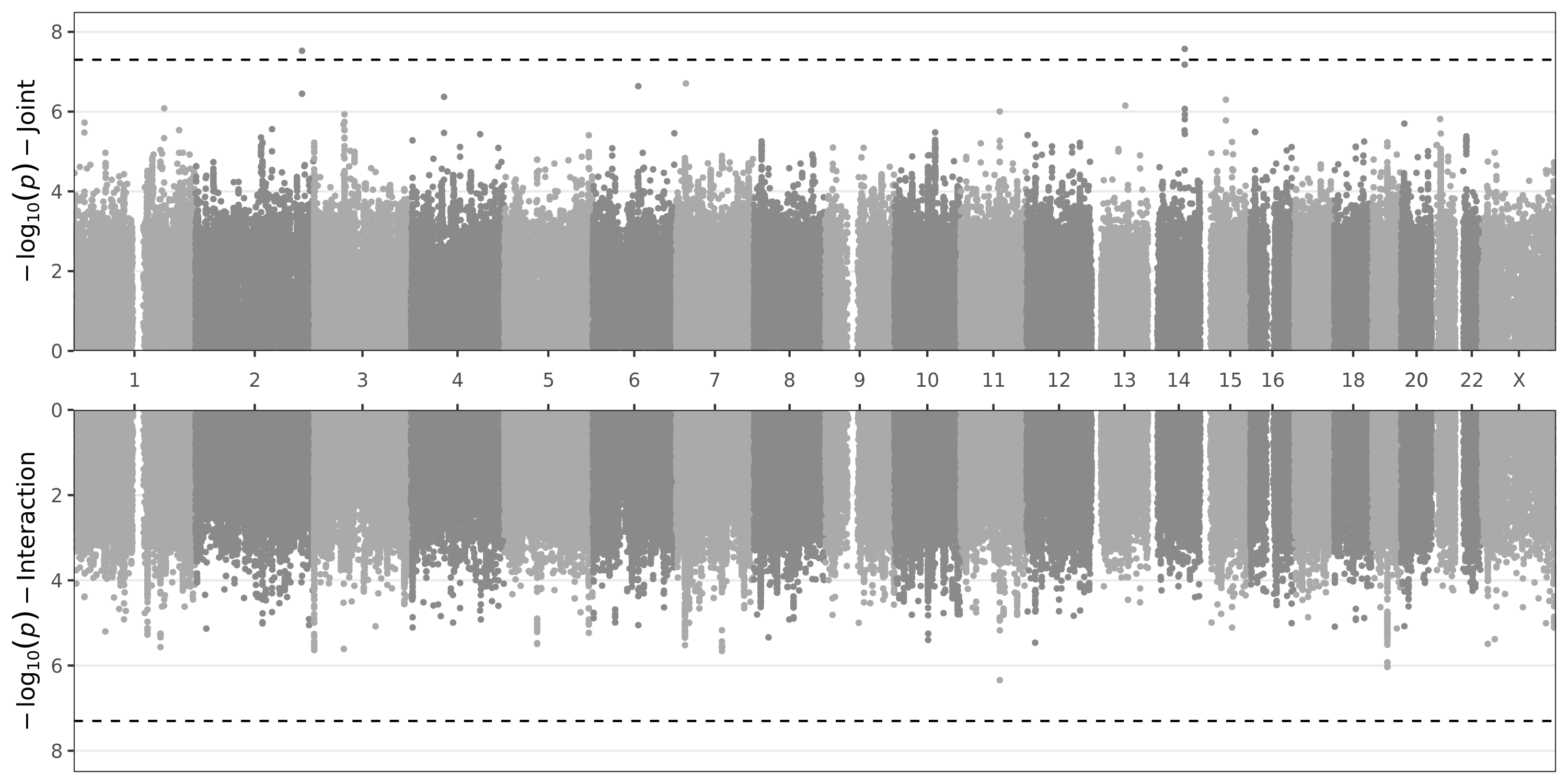
**

**Supp. Fig. S3**: Cardiometabolic analysis joint and interaction analysis results. The Manhattan plot displays association test strengths for the joint (top panel) and interaction (bottom panel) tests as a function of genomic position (*x*-axis).

**
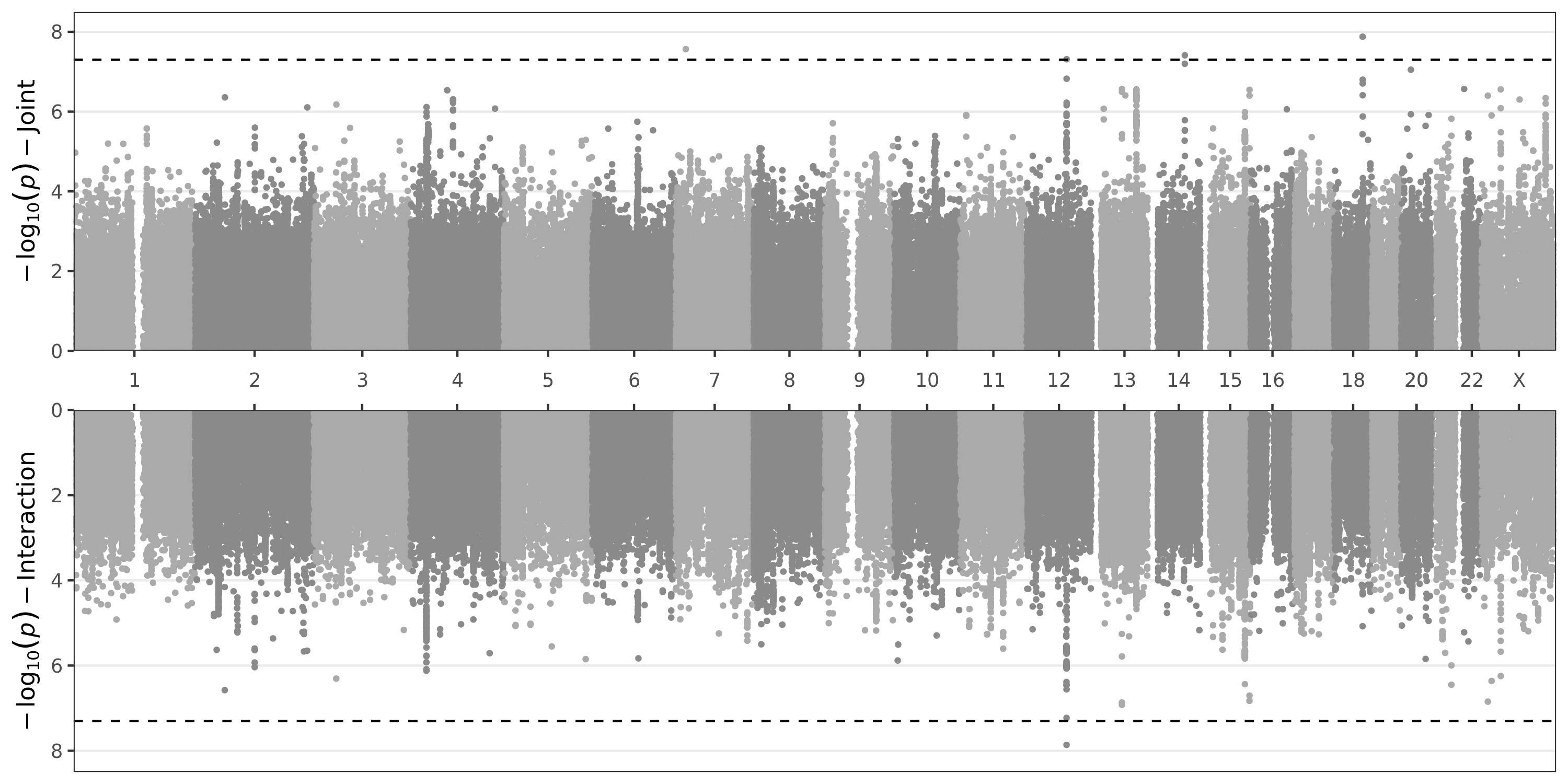
**

**Supp. Fig. S4**: Multiple deprivation index analysis joint and interaction analysis results. The Manhattan plot displays association test strengths for the joint (top panel) and interaction (bottom panel) tests as a function of genomic position (*x*-axis).

**
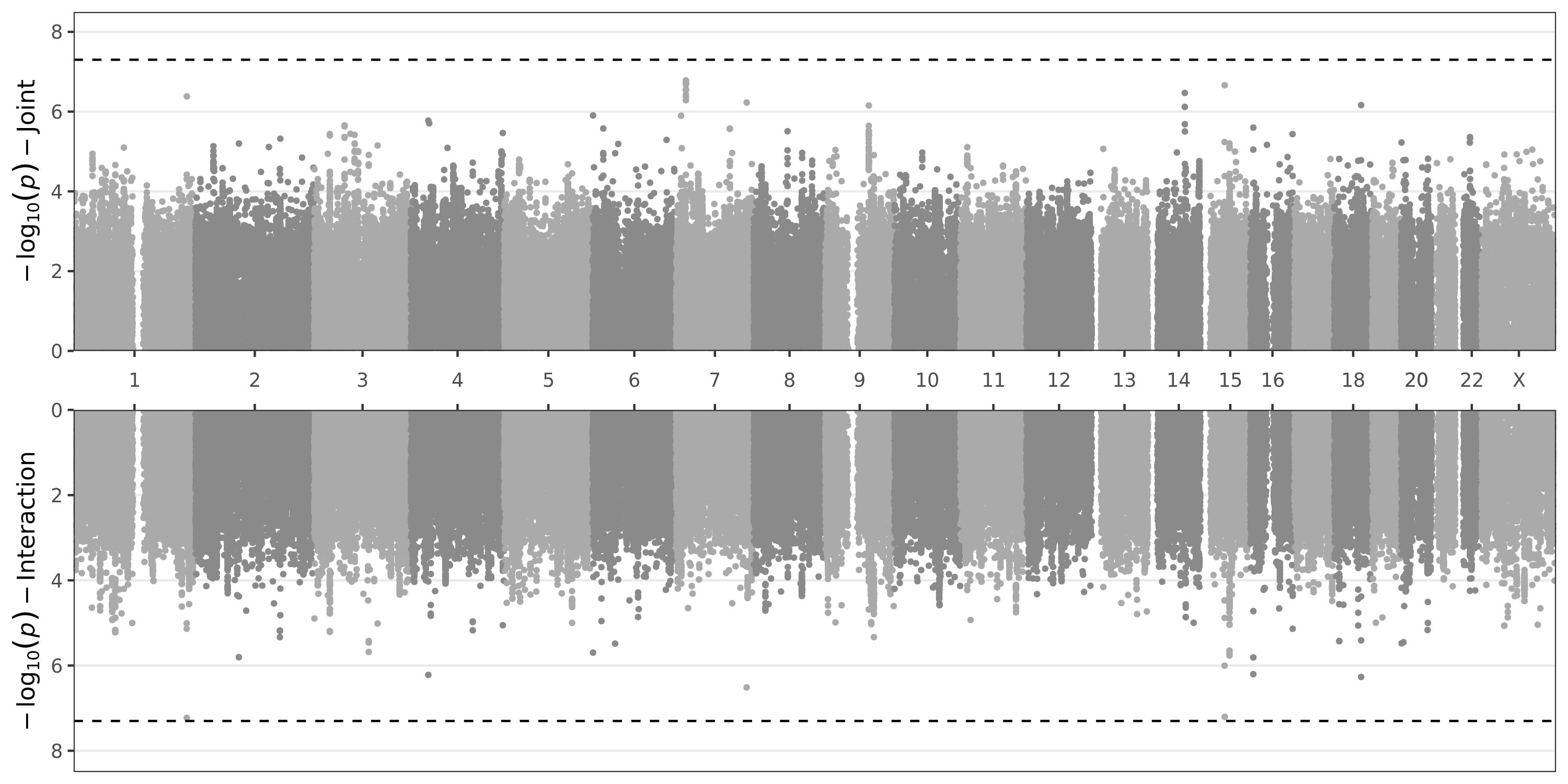
**
